# Does the association between adiposity measures and pre-frailty among older adults vary by social position? Findings from the Tromsø study 2015/2016

**DOI:** 10.1186/s12889-024-18939-3

**Published:** 2024-05-31

**Authors:** Shreeshti Uchai, Lene Frost Andersen, Magne Thoresen, Laila A. Hopstock, Anette Hjartåker

**Affiliations:** 1 Department of Nutrition, Institute of Basic Medical Sciences, University of Oslo, Postbox: 1046, Blindern, Oslo, 0317 Norway; 2 Department of Biostatistics, Institute of Basic Medical Sciences, University of Oslo, Oslo, Norway; 3 Department of Health and Care Sciences, UiT The Arctic University of Norway, Tromsø, Norway

**Keywords:** Pre-frailty, Obesity, Adiposity, Social position, BMI, Waist circumference, Fat mass index, VAT mass, Ageing, Older adults

## Abstract

**Introduction:**

Pre-frailty provides an ideal opportunity to prevent physical frailty and promote healthy ageing. Excess adiposity has been associated with an increased risk of pre-frailty, but limited studies have explored whether the association between adiposity measures and pre-frailty varies by social position.

**Methods:**

We used data from the seventh survey of the Tromsø Study (Tromsø7) conducted in 2015–2016. Our primary sample consisted of 2,945 women and 2,794 men aged ≥ 65 years. Pre-frailty was defined as the presence of one or two of the five frailty components: low grip strength, slow walking speed, exhaustion, unintentional weight loss and low physical activity. Adiposity was defined by body mass index (BMI), waist circumference (WC), fat mass index (FMI) and visceral adipose tissue (VAT) mass. Education and subjective social position were used as measures of social position. Poisson regression with robust variance was used to assess the association between adiposity measures and pre-frailty, and the interaction term between adiposity measures and social position measures were utilised to explore whether the association varied by social position.

**Results:**

In our sample, 28.7% of women and 25.5% of men were pre-frail. We found sub-multiplicative interaction of BMI-defined obesity with education in women and subjective social position in men with respect to development of pre-frailty. No other adiposity measures showed significant variation by education or subjective social position. Regardless of the levels of education or subjective social position, participants with excess adiposity (high BMI, high WC, high FMI and high VAT mass) had a higher risk of pre-frailty compared to those with low adiposity.

**Conclusion:**

We consistently observed that women and men with excess adiposity had a greater risk of pre-frailty than those with low adiposity, with only slight variation by social position. These results emphasize the importance of preventing excess adiposity to promote healthy ageing and prevent frailty among all older adults across social strata.

**Supplementary Information:**

The online version contains supplementary material available at 10.1186/s12889-024-18939-3.

## Introduction

Frailty is a state of age-associated progressive decline in physiological reserve and multisystem dysregulation [1]. This results in decreased coping ability, thus increasing the risk of adverse outcomes, including hospitalization, disability, reduced quality of life and death, even from common external stressors such as minor infections or falls [1–3]. In the context of a rapidly ageing world population [4], frailty presents a major public health challenge. So, preventing frailty and maintaining physical function and independence are integral to healthy ageing [5]. Physical frailty is often characterized by the presence of unintentional weight loss, exhaustion, low physical activity, slow walking speed and low grip strength. Pre-frailty, an intermediate stage often occurring before frailty, provides an optimal opportunity to prevent, delay or even reverse the frailty process and the associated negative consequences, and is thus highly relevant from the perspective of prevention [6, 7].

Growing evidence, including our previous research [8, 9], suggests that high adiposity, assessed by anthropometric measures such as body mass index (BMI) and waist circumference (WC) [10–14], as well as dual-energy X-ray absorptiometry (DXA)-derived measures such as fat mass index (FMI) and visceral adipose tissue (VAT) mass [14–19], is an important risk factor for pre-frailty and frailty. Given the rising obesity epidemic across all age groups [20], this is of major concern. Moreover, various socioeconomic factors such as education and income have been found to have a substantial impact on an individual’s capacity for healthy ageing [21, 22]. The incidence and prevalence of pre-frailty and frailty have been observed to be higher among individuals with lower social position defined by lower level of education [23–27], lower income [24, 25] or manual occupation [27]. This social inequality may be the result of a disproportionate distribution of risk factors for pre-frailty and frailty across different social groups [28]. In addition, social determinants of health interact with a wide range of biological as well as lifestyle-associated risk factors across the life course. This could potentially have a differential impact on the rate of accumulation of health deficits driving the health inequalities across different population groups, where lower social position is often associated with comparatively worse health outcomes, including pre-frailty and frailty. Some studies have reported obesity as one of the key explanatory factors contributing to the socioeconomic differences in frailty [24, 27, 29], while one study suggested an independent effect of obesity and socioeconomic position on frailty [26]. These findings suggest a complex interrelationship of social factors, obesity and the development of frailty.

Most of the studies investigating the association between adiposity measures and frailty status control for different social position-related indicators. However, whether this association varies by social position still remains relatively unexplored [26]. It is important to explore and understand whether and to what extent the difference in social position affects the association between adiposity measures and pre-frailty. The identification of specific social subgroups that are more prone to developing pre-frailty than others can inform the effective planning and implementation of interventions to prevent frailty. It is particularly important in the current context where life expectancy is increasing, but age-associated disorders are also on the rise [30], and health inequality remains as evident as ever [31].

Therefore, in the present study, we explored whether and how the association of adiposity measures with pre-frailty varies by social position among older adults in Tromsø, Norway.

## Methods

### Study population

The present study uses data from the seventh survey of the Tromsø Study (Tromsø7) conducted in 2015–2016. All registered inhabitants in Tromsø municipality aged ≥ 40 years (*N* = 32,591) were invited to Tromsø7 (visit 1). Invitation to extended examinations (visit 2), including DXA scans, required attendance at visit 1 In total, 21,083 individuals aged 40–99 years attended visit 1 (65%). Of these, 9,253 were pre-marked for an invitation to visit 2 In total, 8,346 attended visit 2 (90% of those attending visit 1). Out of these, 3,670 participants underwent whole-body DXA scans. Details about study design, sample recruitment, attendance and data collection have been described elsewhere [32].

Our primary sample comprised 5,739 participants (2,945 women and 2,794 men) aged ≥ 65 years. From the total participants available for analysis (*N* = 21,069), we excluded participants who were < 65 years (*n* = 15,195), had missing information on BMI or WC (*n* = 54), had no information on frailty status (*n* = 21), had missing information on both education and subjective social position (*n* = 5) and were frail (*n* = 55) (Supplementary Fig. 1). For subsample analysis, we included participants with DXA-derived adiposity measures (*n* = 2,191).

### Adiposity measures

#### Anthropometric measures

BMI was calculated as body weight in kilograms divided by the square of height in metres (kg/m^2^). WC was measured using a tape measure to the nearest centimetre at the level of the umbilicus. BMI was categorized as underweight (< 18.5 kg/m^2^), normal (18.5–24.9 kg/m^2^), overweight (25.0–29.9 kg/m^2^) and obese (≥ 30.0 kg/m^2^) [33] and WC was categorized as normal (women ≤ 80 cm, men ≤ 94 cm), moderately high (women 81–88 cm, men 95–102 cm) and high (women > 88 cm, men > 102 cm) [34].

#### DXA-derived measures

Trained technicians conducted DXA scans consistent with the manufacturer’s protocols, using a Lunar Prodigy Advance (GE Medical Systems, Madison, WI, USA) device. The images obtained from DXA scans were inspected and relevant quality corrections were made as per the standardized protocol. The total body fat mass in grams, directly obtained from the DXA measurements, was converted to kilograms and FMI was calculated by dividing the fat mass in kilograms by the square of the height in metres (kg/m^2^). We used FMI as a measure of total adiposity to remove the potential confounding from height. Furthermore, FMI has been suggested to be more accurate in predicting obesity-related health outcomes compared with the more widely used body fat percentage [35]. VAT mass in grams was computed from existing DXA scans using the validated CoreScan application (EnCore version 17.0, GE Healthcare, Madison, WI, USA) [36]. In our subsample, three participants had VAT mass values equal to 0 g and, therefore, in accordance with Lundblad et al. [37], these were manually transformed into the lowest registered value of VAT mass in our study sample (i.e. 2 g).

FMI and VAT mass were categorized into sex-specific tertiles. FMI was categorized in women as low (first tertile [T1]: <9.1 kg/m^2^), medium (second tertile [T2]: 9.1–12.0 kg/m^2^) and high (third tertile [T3]: >12.0 kg/m^2^) and in men as low (T1: <6.5 kg/m^2^), medium (T2: 6.5–8.8 kg/m^2^) and high (T3: >8.8 kg/m^2^). VAT mass was categorized among women as low (T1: <694 g), medium (T2: 694–1,214 g) and high (T3: >1,214 g) and among men as low (T1: <1,286 g), medium (T2: 1,286–2,045 g) and high (T3: >2,045 g).

### Frailty status assessment

Fried et al.’s frailty phenotype definition was utilized to operationalize physical frailty status [1]. Self-reported measures, including unintentional weight loss, exhaustion and low physical activity, and performance-based measures, including walking speed and grip strength, were assessed [1]. Based on the number of frailty indicators present, participants were classified as robust (0), pre-frail (1–2) or frail (≥ 3). As a result of the low frailty prevalence in our sample and the significance of pre-frailty from a public health perspective [6, 7], we focused on pre-frailty as the main outcome in the present study.

The Malnutrition Universal Screening Tool [38] was used to assess involuntary weight loss over the last 6 months. Exhaustion was assessed by the response ‘pretty much’ or ‘very much’ to the question ‘During the last week, have you experienced that everything is a struggle?’ from the Hopkins’ Symptom Checklist-10 [39]. Low physical activity level was defined by the response ‘Reading, watching TV/screen or other sedentary activity’ to the question ‘Describe your exercise and physical exertion in leisure time over the last year’ from the Saltin–Grimby Physical Activity Level Scale [40]. Weakness was defined by sex- and BMI-specific cut-offs for grip strength, as suggested by Fried et al. [1]. Grip strength (kg) was measured using a calibrated Jamar + Digital Dynamometer (notch 2; Patterson Medical, Warrenville, IL, USA) following the Southampton protocol procedures [41]. Walking speed was measured using timed walking speed from the Short Physical Performance Battery (SPPB) [42, 43], in which the fastest time out of two walks was selected and converted to seconds per 15 feet from seconds per 4 m, with sex- and height-adjusted cut-offs, according to Fried et al. [1].

We describe each frailty indicator used in the present study and its comparison with Fried et al.’s definition in detail in Supplementary Table 1.

### Indicators of social position

Education and subjective social position were utilized as objective and subjective measures of social position, respectively. Education was categorized as primary/partly secondary (schooling up to 10 years), upper secondary (minimum of 3 years), short tertiary (college/university < 4 years) or long tertiary (college/university ≥ 4 years) based on the response to the question ‘What is the highest level of education you have completed?’ [44]. We reorganized these responses to form two categories depicting the length of education based on the reported highest level of education, where ≤ 10 years of education represented primary/partly secondary education, and > 10 years of education included upper secondary and tertiary education levels.

We assessed subjective social position from the statement ‘I consider my occupation to have the following social status (if you are currently out of work, think about your latest occupation)’, rated using a five-level scale (very high; fairly high; middle; fairly low; very low). The upper two responses ‘very high’ and ‘fairly high’ and the bottom two responses ‘fairly low’ and ‘very low’ were merged to form ‘high’ and ‘low’ categories, respectively, and the response ‘middle’ was categorized as ‘medium’. Due to a low number of individuals in the ‘low’ category, the ‘low’ category was excluded in the stratified analysis.

### Covariates

The covariates were selected based on the existing literature. Sociodemographic characteristics included age, sex and marital/cohabitation status. Self-reported lifestyle factors included smoking (current, former or never smoker) and alcohol intake (never-drinker, infrequent drinker [≤ 2–4 times/month] and frequent drinker [≥ 2–3 times/week]). Self-perceived health was categorized as ‘good’ for the responses ‘very good’ or ‘good’ and ‘poor’ for ‘neither good nor bad’ or ‘bad’ or ‘very bad’ to the question ‘How do you consider your health in general?’. Comorbidity was classified as ‘no comorbidity’ or ‘comorbidity’ based on self-reported presence (former and/or current) of two or more of the following diseases: coronary heart disease (angina pectoris/myocardial infarction), stroke, diabetes, cancer, hypertension, arthritis, kidney disease and pulmonary disease (asthma/chronic bronchitis/emphysema).

### Statistical analysis

The participants’ characteristics, including sociodemographic and lifestyle factors and adiposity measures, across robust and pre-frail groups, are presented using proportion and count for categorical variables and mean and standard deviation (SD) for continuous variables. χ^2^ tests were used to assess differences between robust and pre-frail groups for categorical variables and Student’s *t*-tests for continuous variables.

Poisson regression with robust standard errors was used to assess the cross-sectional association between different adiposity measures and pre-frailty, and relative risk (RR) with a 95% confidence interval (CI) is reported. Absolute risk in the form of risk difference (RD) with 95% CI is also presented to complement the RR estimates [45]. The RD estimates were calculated using the ‘binreg’ function in STATA with the RD option, which fits generalized linear models for the binomial family using an identity link. We ran separate models for BMI, WC, FMI and VAT mass. Each adiposity measure was entered in the fitted models in their predefined categorical forms. The models were adjusted for age, smoking status, alcohol intake, comorbidity, marital/cohabitation status, education and self-perceived health. The underweight group from the BMI categories was removed in all analyses, except descriptive, as a result of the low number of observations.

To explore whether the association between adiposity and pre-frailty varied by education or subjective social position, we tested for effect modification by introducing an interaction term for length of education/ subjective social position and adiposity measure in the adjusted model and assessed its statistical significance. This was carried out separately for each adiposity measure, and the stratified RR estimates, along with the interaction term are presented. In addition, RR and RD estimates for different combinations of adiposity measures and social position, with one common reference group, were computed using the results from the model with the interaction term. For example, for the model analysing the effect of education on the association between BMI and pre-frailty, the RR estimates were reported for the categories: normal BMI and > 10 years of education (reference); normal BMI and ≤ 10 years of education; overweight and > 10 years of education; overweight and ≤ 10 years of education; and obesity and > 10 years of education; and obesity and ≤ 10 years of education. For the models with significant interaction terms, these combined estimates are presented graphically, whereas the rest are presented in detail along with RD estimates in the supplementary section. For additional supplementary analyses, we explored how different adiposity measures were distributed across different social groups, characterized by education and subjective social position. We also explored how the participants’ characteristics varied across robust and pre-frail groups with scores 1 and 2. Furthermore, we also assessed frequencies of individual frailty indicators underlying pre-frailty.

As women and men significantly vary in terms of their body composition and sex-associated norms and roles [46, 47], all our analyses are sex-stratified. STATA 16 was used for all analyses [48], and statistical significance was set at *P* < 0.05.

## Results

### Population characteristics

Table 1 displays the sex-stratified characteristics of the study population by pre-frailty status. In total, 27.1% were pre-frail with a mean age of 73.3 years, significantly higher than the robust population (71.6 years). A total of 51.3% were women, and when stratified by sex, 28.7% of women and 25.5% of men were pre-frail. The pre-frail women and men differed significantly from the robust ones in terms of smoking status, alcohol intake, marital/cohabitation status, self-perceived health and comorbidity. Furthermore, in pre-frail women (58.6% versus 45.2%) and men (38.7% versus 32.0%), a significantly higher proportion had ≤ 10 years of education. In pre-frail women (30.5% versus 33.4%) and men (44.5% versus 50.2%), a lower proportion perceived their social position as high; however, the difference was only significant among men. Both pre-frail women and men had significantly higher mean BMI, WC, FMI and VAT mass than their robust counterparts (Table 1).

When further comparing the participant’s characteristics across the pre-frail group with 1 and 2 frailty indicators, we observed the latter group to be older with a higher proportion of comorbidities, poor self-reported health, obesity, high WC, and higher FMI and VAT mass (Supplementary Table 2).

**Table 1:** Participants’ characteristics by pre-frailty status

	Women (*n* = 2,945)			Men (*n* = 2,794)		
	Frailty status			Frailty status		
	Robust (%) (*n*)	Pre-frail (%) (*n*)	*P* value	Robust (%) (*n*)	Pre-frail (%) (*n*)	*P* value
	71.3 (2,099)	28.7 (846)		74.5 (2,083)	25.5 (711)	
**Age, years, mean (SD)**	71.5 (5.6)	73.6 (6.6)	< 0.001^a^	71.6 (5.5)	72.9 (6.0)	< 0.001^a^
**Smoking status**						
Current smokers	10.4 (214)	14.0 (116)		8.2 (168)	15.6 (110)	
Former smokers	46.9 (969)	47.2 (393)	0.012	58.3 (1201)	57.5 (405)	< 0.001
Never	42.7 (883)	38.8 (323)		33.5 (690)	26.9 (189)	
**Alcohol**						
Frequent drinkers	26.7 (552)	18.4 (153)		35.3 (729)	27.5 (194)	
Infrequent drinkers	59.5 (1229)	57.4 (478)	< 0.001	56.3 (1165)	64.2 (453)	< 0.001
Never/Abstaining	13.8 (286)	24.2 (202)		8.4 (173)	8.4 (59)	
**Married/Cohabiting**						
Married/Cohabiting	58.7 (1232)	48.2 (408)	< 0.001	80.6 (1679)	75.4 (536)	0.003
Living alone	41.3 (867)	51.8 (438)		19.4 (404)	24.6 (175)	
**Self-perceived health**						
Good	68.2 (1404)	41.4 (344)	< 0.001	69.8 (1440)	46.4 (327)	< 0.001
Poor	31.8 (654)	58.6 (487)		30.2 (624)	53.6 (378)	
**Comorbidity**						
No comorbidity	61.1 (1275)	46.4 (389)	< 0.001	61.9 (1287)	48.2 (341)	< 0.001
Comorbidity	38.9 (811)	53.6 (450)		38.1 (791)	51.8 (367)	
**Education**						
> 10 years of education	54.8 (1142)	41.4 (347)		68.0 (1405)	61.3 (433)	
≤ 10 years of education	45.2 (942)	58.6 (492)	< 0.001	32.0 (661)	38.7 (273)	< 0.001
**Subjective social position**						
High	33.4 (666)	30.5 (232)	0.304	50.2 (1009)	44.5 (299)	0.014
Medium	59.7 (1188)	62.7 (478)		46.7 (940)	50.9 (342)	
Low	6.9 (137)	6.8 (52)		3.1 (62)	4.6 (31)	
**Adiposity measures**						
**BMI, kg/m**^**2**^, **mean (SD)**	26.8 (4.4)	28.2 (5.7)	< 0.001^a^	27.3 (3.5)	28.2 (4.5)	< 0.001^a^
Underweight	1.0 (20)	2.0 (17)		0.1 (2)	0.4 (3)	
Normal	35.4 (743)	25.2 (213)	< 0.001	26.0 (541)	23.1 (164)	< 0.001
Overweight	43.0 (902)	38.5 (326)		53.7 (1118)	44.7 (318)	
Obesity	20.6 (434)	34.3 (290)		20.2 (422)	31.8 (226)	
**WC, cm, mean (SD)**	*91.7 (11.7)*	*95.6 (13.9)*	< 0.001^a^	*100.4 (10.1)*	*103.9 (12.3)*	< 0.001^a^
Normal	17.9 (376)	13.0 (110)		28.2 (587)	21.4 (152)	
Moderately high	23.5 (493)	17.6 (149)	< 0.001	32.3 (673)	25.7 (183)	< 0.001
High	58.6 (1230)	69.4 (587)		39.5 (823)	52.9 (376)	
	***n*** **= 1,282**			***n*** **= 911**		
**FMI, kg/m**^**2**^, **mean (SD)**	*10.4 (3.2)*	*11.7 (4.1)*	< 0.001^a^	*7.6 (2.5)*	*8.3 (2.9)*	< 0.001^a^
**FMI tertiles**						
Low (T1)	36.2 (342)	25.6 (86)		35.7 (243)	26.5 (61)	
Medium (T2)	35.3 (334)	27.7 (93)	< 0.001	32.3 (220)	36.5 (84)	0.039
High (T3)	28.5 (270)	46.7 (157)		32.0 (218)	37.0 (85)	
**VAT mass, g, mean (SD)**	983 (594)	1178 (733)	< 0.001^a^	1685 (827)	1888 (988)	0.002^a^
**VAT mass tertiles**						
Low (T1)	35.4 (335)	27.6 (93)		35.3 (241)	27.8 (64)	
Medium (T2)	34.4 (325)	30.6 (103)	< 0.001	33.8 (231)	31.8 (73)	0.020
High (T3)	30.2 (286)	41.8 (141)		30.9 (211)	40.4 (93)	

Values are mean values (standard deviations) or percentages (numbers)

*P* value: χ^2^ test for categorical variables; ^a^*P* value: Student’s *t*-test for continuous variables

BMI, body mass index; FMI, fat mass index; SD, standard deviation; VAT, visceral adipose tissue; WC, waist circumference

T1: first tertile; T2: second tertile; T3: third tertile

*BMI categories* Underweight: <18.5 kg/m^2^, Normal: 18.5–24.9 kg/m^2^, Overweight: 25.0–29.9 kg/m^2^, Obesity: ≥30 kg/m^2^

*FMI categories* Low (T1): women < 9.1 kg/m^2^; men < 6.5 kg/m^2^, Medium (T2): women 9.1–12.0 kg/m^2^; men 6.5–8.8 kg/m^2^, High (T3): women > 12.0 kg/m^2^; men > 8.8 kg/m^2^

*WC categories* Low: women ≤ 80 cm; men ≤ 94 cm, Moderately high: women 81–88 cm; men 95–102 cm, High: women > 88 cm; men > 102 cm

*VAT categories* Low (T1): women < 694 g; men < 1286 g, Medium (T2): women 694–1,214 g; men 1,286–2,045 g, High (T3): women > 1,214 g; men > 2,045 g

In the supplementary analysis (Supplementary Table 3), both women and men with a short length of education had a significantly higher proportion of high BMI, WC, FMI and VAT mass compared with those with a long length of education; however, in men, this was only statistically significant for obesity defined by BMI. Among women, participants with lower subjective social position had statistically significantly higher proportions of high BMI, WC and FMI but not VAT mass. No significant difference in the distribution of adiposity measures was found in men with low versus high subjective social position.

### Adiposity and pre-frailty

Table 2 depicts the association between different adiposity measures and pre-frailty, when adjusted for covariates. Women with general (high BMI, high FMI) or abdominal (high WC, high VAT mass) adiposity were found to have increased risk of pre-frailty when compared with those without adiposity. Similarly, men with high BMI, high WC or high VAT mass had an increased risk of having pre-frailty. Meanwhile, the association between high FMI and pre-frailty was not significant among men.

**Table 2:** Association between adiposity measures and pre-frailty

	Women (*n* = 2,945)		Men (*n* = 2,794)	
	RR (95% CI)	RD (95% CI)	RR (95% CI)	RD (95% CI)
**BMI categories**				
Normal	Ref	Ref	Ref	Ref
Overweight	1.07 (0.92–1.24)	0.02 (− 0.02 to 0.05)	0.99 (0.84–1.17)	0.01 (− 0.03 to 0.04)
Obesity	**1.53 (1.32–1.78)**	**0.14 (0.09–0.18)**	**1.49 (1.25–1.78)**	**0.11 (0.07–0.16)**
**WC categories**				
Normal	Ref	Ref	Ref	Ref
Moderately high	0.97 (0.78–1.19)	0.00 (− 0.04 to 0.04)	1.03 (0.85–1.24)	0.00 (− 0.04 to 0.04)
High	**1.19 (1.00–1.41)**	**0.05 (0.01–0.09)**	**1.39 (1.18–1.65)**	**0.08 (0.04–0.12)**
**FMI tertiles**	*n *= 1,282		* n* = 911	
Low (T1)	Ref	Ref	Ref	Ref
Medium (T2)	1.04 (0.79–1.36)	0.01 (− 0.04 to 0.06)	1.32 (0.99–1.76)	0.06 (− 0.01 to 0.13)
High (T3)	**1.61 (1.26–2.05)**	**0.13 (0.07–0.19)**	1.32 (0.98–1.76)	0.05 (− 0.01 to 0.11)
**VAT mass tertiles**				
Low (T1)	Ref	Ref	Ref	Ref
Medium (T2)	1.01 (0.79–1.29)	−0.01 (− 0.06 to 0.04)	1.13 (0.84–1.52)	0.02 (− 0.03 to 0.08)
High (T3)	**1.29 (1.01–1.64)**	**0.07 (0.01–0.13)**	**1.35 (1.02–1.79)**	**0.07 (0.00–0.14)**

CI, confidence interval; RD, risk difference; RR, risk ratio

Adjusted for age, smoking status, alcohol intake status, comorbidity, marital/cohabitation status, education and self-perceived health

BMI, body mass index; FMI, fat mass index; SD, standard deviation; VAT, visceral adipose tissue; WC, waist circumference

T1: first tertile; T2: second tertile; T3: third tertile

*BMI categories* Underweight: <18.5 kg/m^2^, Normal: 18.5–24.9 kg/m^2^, Overweight: 25.0–29.9 kg/m^2^, Obesity: ≥30 kg/m^2^

*FMI categories* Low (T1): women < 9.1 kg/m^2^; men < 6.5 kg/m^2^, Medium (T2): women 9.1–12.0 kg/m^2^; men 6.5–8.8 kg/m^2^, High (T3): women > 12.0 kg/m^2^; men > 8.8 kg/m^2^

*WC categories* Normal: women ≤ 80 cm; men ≤ 94 cm, Moderately high: women 81–88 cm; men 95–102 cm, High: women > 88 cm; men > 102 cm

*VAT categories* Low (T1): women < 694 g; men < 1,286 g, Medium (T2): women 694–1,214 g; men 1,286–2,045 g, High (T3): women > 1,214 g; men > 2,045 g

### Adiposity, pre-frailty and effect modification by measures of social position

#### By education

Table 3 displays the association between various adiposity measures and pre-frailty in both women and men, stratified by length of education. We observed a significant modification of the association between BMI and pre-frailty by education among women. The interaction effect was sub-multiplicative, suggesting that the combined effect of having both ≤ 10 years of education and high BMI (overweight or obesity) is less than what would be expected based on the product of the individual effects of having ≤ 10 years of education and high BMI (overweight or obesity).

**Fig. 1:**
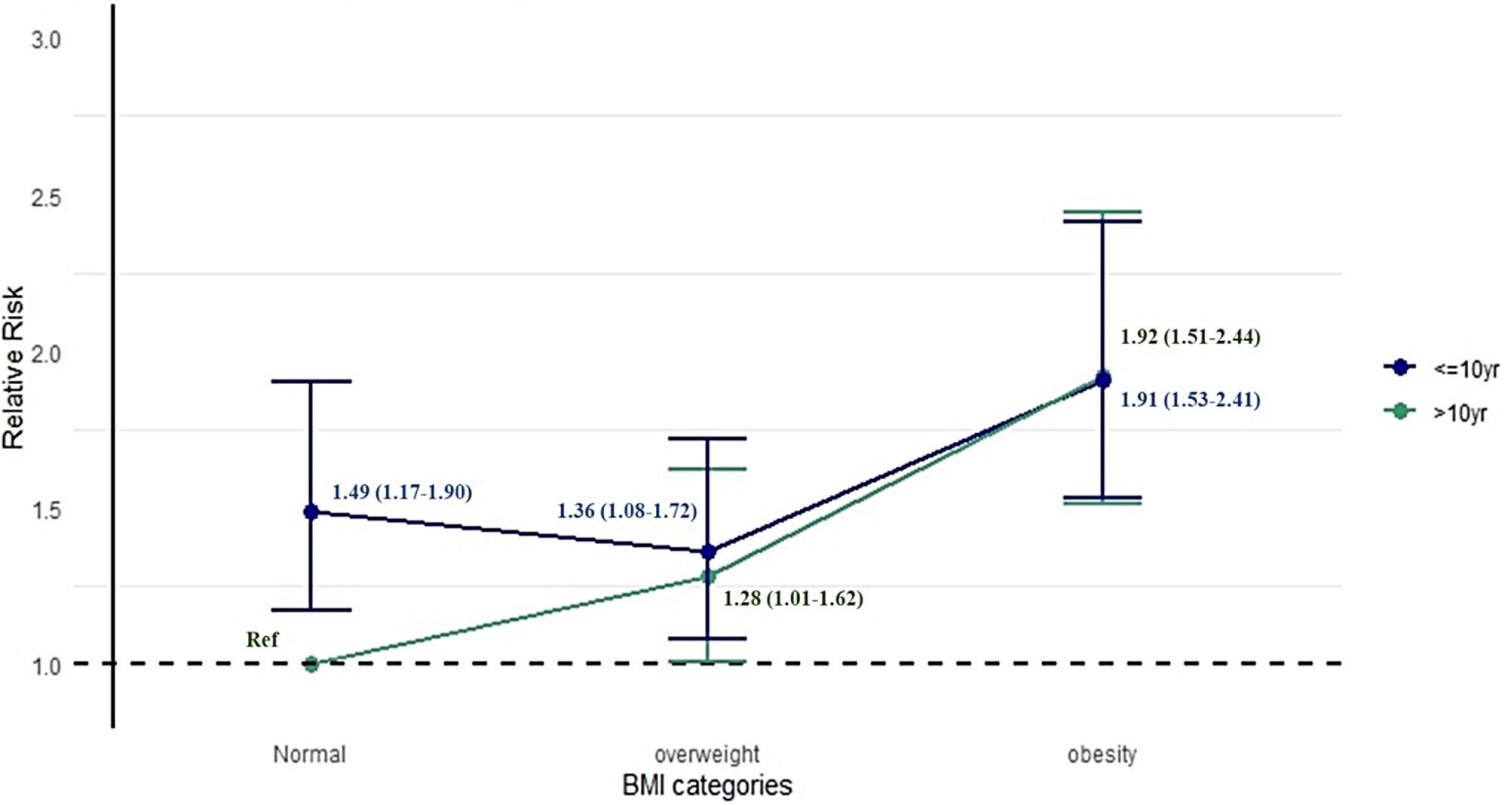
Association between different combinations of body mass index (BMI) categories and pre-frailty by education among women, with normal BMI and > 10 years of education as the reference group, expressed as relative risk (RR) with 95% confidence intervals (CIs), adjusted for age, smoking status, alcohol intake status, comorbidity and self-perceived health

Compared with women with normal BMI, the RR (95% CI) of pre-frailty among women with overweight or obesity in the > 10 years education stratum was 1.28 (1.01–1.62) and 1.92 (1.51–2.44), respectively. Meanwhile, in the ≤ 10 years education stratum, the RR of pre-frailty among women with overweight or obesity was 0.91 (0.76–1.11) and 1.28 (1.07–1.54), respectively, compared with those with normal BMI. Furthermore, when compared across different combinations of BMI categories and length of education among women, with normal BMI and > 10 years of education as the reference group, the risk of pre-frailty was 1.49 (1.17–1.90) among women with normal BMI and ≤ 10 years of education, 1.92 (1.51–2.44) among women with obesity and > 10 years of education and 1.91 (1.53–2.41) among women with obesity and ≤ 10 years of education (Fig. 1 and Supplementary Table 4).

There was also an indication of a sub-multiplicative interaction effect between FMI and length of education among women with respect to the risk of pre-frailty, but this was observed only for medium and not for high FMI (Table 3). No statistically significant interaction was observed between length of education and abdominal adiposity (i.e., WC and VAT mass) in women. In men, no statistically significant interaction was detected between education and any adiposity measures.

#### By subjective social position

Table 3 displays the association between various adiposity measures and pre-frailty in both women and men, stratified by subjective social position. Among women, there were no significant modifications of the association between any of the adiposity measures and pre-frailty by subjective social position.

Among men, a significant modification of the association between BMI-defined obesity and pre-frailty by subjective social position was observed, with no notable effect detected for the rest of the adiposity measures. Specifically, a sub-multiplicative interaction was detected between obesity and medium subjective social position. This suggests that the combined effect of having medium subjective social position and obesity is lower than the product of the individual effects of medium subjective social position and obesity.

**Fig. 2:**
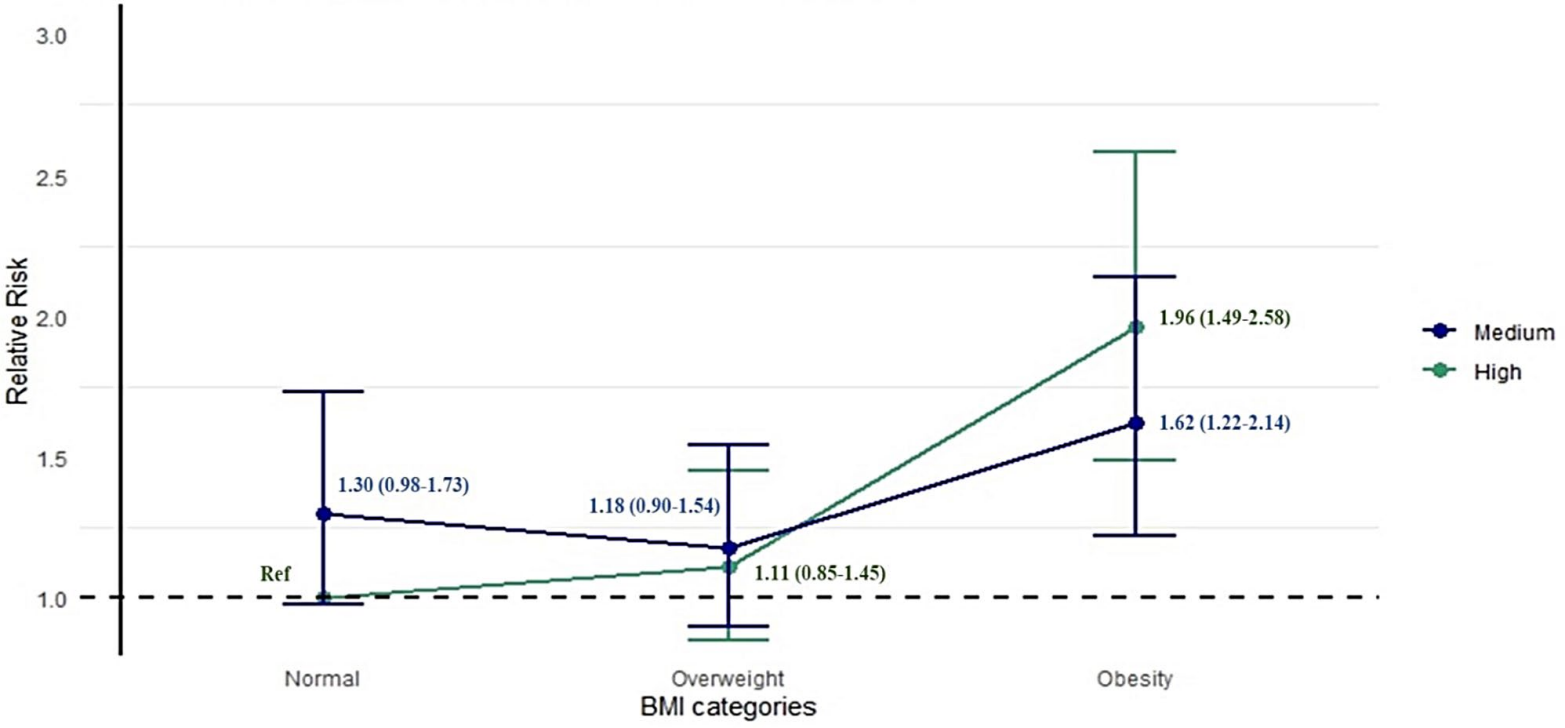
Association between different combinations of body mass index (BMI) categories and pre-frailty by subjective social position among men, with normal BMI and high subjective social position as the reference group, expressed as relative risk (RR) with 95% confidence intervals (CIs), adjusted for age, smoking status, alcohol intake status, comorbidity and self-perceived health

In the high subjective social position stratum, when compared with men with normal BMI, the RR (95% CI) of pre-frailty among men with overweight or obesity was 1.11 (0.85–1.45) and 1.96 (1.49–2.58), respectively. Meanwhile, in the medium subjective social position stratum, the risk of pre-frailty among men with overweight or obesity compared with normal BMI was 0.91 (0.72–1.13) and 1.25 (0.98–1.59), respectively. Furthermore, when compared across different combinations of BMI categories and subjective social position among men, with normal BMI and high subjective social position as the reference group, the risk of pre-frailty was 1.30 (0.98–1.73) among those with normal BMI and medium subjective social position, 1.62 (1.22–2.14) among those with obesity who have medium subjective social position and 1.96 (1.49–2.58) among those with obesity who have high subjective social position (Fig. 2 and Supplementary Table 5).

**Table 3:** Association between adiposity measures and pre-frailty by education and subjective social position

	Education			Subjective social position	
	Women	Men		Women	Men
	RR (95% CI)	RR (95% CI)		RR (95% CI)	RR (95% CI)
**BMI**	*n* = 2,886	*n* = 2,767	**BMI**	*n* = 2,533	*n* = 2,585
**> 10 years**			**High**		
Normal BMI	Ref	Ref	Normal BMI	Ref	Ref
Overweight	1.28 (1.01–1.62)	1.02 (0.82–1.26)	Overweight	1.08 (0.82– 1.43)	1.11 (0.85–1.45)
Obesity	1.92 (1.51–2.44)	1.56 (1.25–1.95)	Obesity	1.70 (1.29–2.22)	1.96 (1.49–2.58)
**≤ 10 years**			**Medium**		
Normal BMI	Ref	Ref	Normal BMI	Ref	Ref
Overweight	0.91 (0.76–1.11)	0.95 (0.73–1.22)	Overweight	1.13 (0.93–1.38)	0.91 (0.72–1.13)
Obesity	1.28 (1.07–1.54)	1.38 (1.06–1.80)	Obesity	1.59 (1.30–1.95)	1.25 (0.98–1.59)
**Multiplicative interaction**			**Multiplicative interaction**		
Overweight *≤10 years	**0.71 (0.53–0.96)**	0.93 (0.67–1.29)	Overweight *medium	1.05 (0.74–1.48)	0.82 (0.58–1.16)
Obesity *≤10 years	**0.67 (0.50–0.90)**	0.88 (0.63–1.24)	Obesity *medium	0.94 (0.67–1.30)	**0.64 (0.45–0.91)**
**WC**	*n* = 2,923	*n* = 2,772	**WC**	*n* = 2,564	*n* = 2,590
**> 10 years**			**High**		
Normal WC	Ref	Ref	Normal WC	Ref	Ref
Moderate WC	0.90 (0.64–1.24)	0.96 (0.75–1.23)	Moderate WC	0.96 (0.66–1.41)	1.05 (0.78–1.42)
High WC	1.29 (1.00–1.67)	1.37 (1.11–1.69)	High WC	1.18 (0.87–1.59)	1.57 (1.21–2.04)
**≤ 10 years**			**Medium**		
Normal WC	Ref	Ref	Normal WC	Ref	Ref
Moderate WC	1.01 (0.77–1.32)	1.14 (0.85–1.53)	Moderate WC	0.96 (0.72–1.29)	1.02 (0.78–1.33)
High WC	1.11 (0.89–1.38)	1.44 (1.11–1.88)	High WC	1.30 (1.02–1.66)	1.28 (1.02–1.62)
**Multiplicative interaction**			**Multiplicative interaction**		
Moderate WC *≤10 years	1.13 (0.74–1.72)	1.18 (0.81–1.73)	Moderate WC *medium	0.99 (0.62–1.60)	0.97 (0.65–1.44)
High WC *≤10 years	0.86 (0.61–1.21)	1.05 (0.75–1.47)	High WC *medium	1.10 (0.75–1.61)	0.82 (0.58–1.15)
**FMI**	*n* = 1,278	*n* = 905	**FMI**	*n* = 1,137	*n* = 850
**> 10 years**			**High**		
Low FMI	Ref	Ref	Low FMI	Ref	Ref
Medium FMI	1.37 (0.94–1.99)	1.32 (0.91–1.91)	Medium FMI	0.98 (0.63–1.52)	1.53 (0.96–2.46)
High FMI	1.82 (1.27–2.63)	1.16 (0.78–1.72)	High FMI	1.38 (0.93–2.04)	1.42 (0.88–2.29)
**≤ 10 years**			**Medium**		
Low FMI	Ref	Ref	Low FMI	Ref	Ref
Medium FMI	0.78 (0.54–1.13)	1.29 (0.82–2.03)	Medium FMI	1.38 (0.96–2.00)	1.15 (0.78–1.70)
High FMI	1.39 (1.03–1.88)	1.53 (1.01–2.34)	High FMI	1.93 (1.37–2.73)	1.25 (0.85–1.83)
**Multiplicative interaction**			**Multiplicative interaction**		
Medium FMI * ≤10 years	**0.57 (0.34–0.97)**	0.97 (0.54–1.74)	Medium FMI * medium	1.41 (0.80–2.51)	0.75 (0.41–1.38)
High FMI * ≤10 years	0.76 (0.48–1.21)	1.31 (0.74–2.31)	High FMI * medium	1.40 (0.84–2.33)	0.88 (0.48–1.60)
**VAT mass**	*n* = 1,279	*n* = 907	**VAT mass**	*n* = 1,138	*n* = 852
**> 10 years**			**High**		
Low VAT mass	Ref	Ref	Low VAT mass	Ref	Ref
Medium VAT mass	1.07 (0.74–1.54)	1.08 (0.73–1.59)	Medium VAT mass	1.08 (0.70–1.66)	1.49 (0.91–2.42)
High VAT mass	1.45 (1.02–2.04)	1.27 (0.88–1.83)	High VAT mass	1.26 (0.85–1.87)	1.46 (0.90–2.36)
**≤ 10 years**			**Medium**		
Low VAT mass	**Ref**	Ref	Low VAT mass	Ref	Ref
Medium VAT mass	0.93 (0.66–1.29)	1.21 (0.77–1.90)	Medium VAT mass	1.11 (0.80–1.55)	0.83 (0.54–1.26)
High VAT mass	1.15 (0.84–1.58)	1.48 (0.98–2.23)	High VAT mass	1.47 (1.07–2.03)	1.29 (0.90–1.84)
**Multiplicative interaction**			**Multiplicative interaction**		
Medium VAT mass * ≤10 years	0.86 (0.53–1.41)	1.12 (0.62–2.02)	Medium VAT mass * medium	1.03 (0.59–1.77)	0.56 (0.29–1.05)
High VAT mass * ≤10 years	0.79 (0.51–1.25)	1.16 (0.68–1.99)	High VAT mass * medium	1.17 (0.71–1.93)	0.88 (0.49–1.59)

*, multiplicative interaction; CI, confidence interval; RR, risk ratio

Adjusted for age, smoking status, alcohol intake status, comorbidity, and self-perceived health

BMI, body mass index; FMI, fat mass index; SD, standard deviation; VAT, visceral adipose tissue; WC, waist circumference

T1: first tertile; T2: second tertile; T3: third tertile

*BMI categories* Underweight: <18.5 kg/m^2^, Normal: 18.5–24.9 kg/m^2^, Overweight: 25.0–29.9 kg/m^2^, Obesity: ≥30 kg/m^2^

*FMI categories* Low (T1): women < 9.1 kg/m^2^; men < 6.5 kg/m^2^, Medium (T2): women 9.1–12.0 kg/m^2^; men 6.5–8.8 kg/m^2^, High (T3): women > 12.0 kg/m^2^; men > 8.8 kg/m^2^

*WC categories* Normal: women ≤ 80 cm; men ≤ 94 cm, Moderately high: women 81–88 cm; men 95–102 cm, High: women > 88 cm; men > 102 cm

*VAT categories* Low (T1): women < 694 g; men < 1,286 g, Medium (T2): women 694–1,214 g; men 1,286–2,045 g, High (T3): women > 1,214 g; men > 2,045 g

## Discussion

Our study explored whether the association between adiposity measures and pre-frailty varied by social factors, operationalized by education and subjective social position, in 5,739 older adults from a general population residing in Tromsø, Norway. We found that higher levels of adiposity, including BMI-defined obesity, high WC, high FMI and high VAT mass, among women and men were associated with a heightened risk of pre-frailty compared with those with normal adiposity levels. This was observed consistently within the strata of education and subjective social position.

In total, 27.1% of our study participants were pre-frail, which is comparatively lower than the pooled prevalence estimates reported in a recent review by O’Caoimh et al. [49]. We observed a significant effect modification by education in the association between BMI-defined obesity and pre-frailty among women, suggesting a stronger association between obesity and pre-frailty among women with > 10 years of education compared with women who had ≤ 10 years of education. However, when compared with those with normal BMI and > 10 years of education, the risk of pre-frailty was higher among women with obesity regardless of length of education and only minor variations were observed. Noticeably, women with normal BMI and ≤ 10 years of education had a higher risk of pre-frailty compared with their counterparts with a higher education. In line with our findings, a cross-sectional study conducted among 3,005 adults aged 57–85 years reported a similar pattern in the association between BMI categories and the level of an inflammatory marker, C-reactive protein [50]. Inflammatory markers have been closely linked with the development of frailty [51]. This study reported that women and men with lower BMIs were the ones who fared worse with increased inflammatory markers if they had a low level of education, whereas no significant education gradient was observed among those who had severe obesity [50]. Meanwhile, a cross-sectional study carried out among 2,319 Spanish adults aged ≥ 50 years reported a homogeneous effect of obesity on frailty status at all educational levels [26]. When stratified by subjective social position rather than length of education, we observed a significant effect modification by subjective social position on the association between BMI-defined obesity and pre-frailty among men, suggesting a stronger association between pre-frailty and obesity in men with higher subjective social position than those with lower subjective social position. However, men with obesity exhibited an increased risk of pre-frailty compared with the normal BMI group, regardless of the level of subjective social position. Notably, men with normal BMI and medium subjective social position displayed a higher risk of pre-frailty compared with their counterparts in the high social position category. To our knowledge, no studies investigating the effect of subjective social position on the association between adiposity and pre-frailty have been carried out.

Social position has been closely linked to various health outcomes [52–54]. Individuals with low social position often tend to have an unfavourable accumulation of risk factors, including excess adiposity, and limited access to resources, which increases their vulnerability to adverse health outcomes [28, 50, 52, 54, 55]. However, we did not observe a significantly stronger association between adiposity measures and pre-frailty among those with lower social position when compared to those with higher social position. In contrast, our findings indicated a slightly stronger association between BMI-defined obesity and pre-frailty in women with > 10 years of education and men with higher subjective social position. Some studies have suggested this pattern may stem from risk saturation [50, 56]. This implies that as individuals with low social position are already exposed to multiple risk factors, excess adiposity might be only one of many contributors and might not have as significant impact on their health compared to individuals in higher social strata, who have been exposed to fewer competing risk factors [50, 56]. However, it is important to note that the effect modification was not statistically significant for the association between pre-frailty and any other measures of excess adiposity, except BMI, in both women and men. Further, we would like to re-emphasize that pre-frailty risk remained elevated among those with excess adiposity across all social strata, underscoring the importance of addressing excess adiposity. While these findings hold potential for generalizability to older populations from other parts of Norway and Scandinavian countries with similar living conditions, we should be cautious about the generalizability of these findings to regions with more varied access to healthcare since Norway has a robust public healthcare system, universal health coverage and one of the lowest rates of unmet medical care needs in Europe [57].

One of the strengths of our study is that, in addition to using BMI and WC as measures of adiposity, we also utilized DXA-derived adiposity metrics, i.e. FMI and VAT mass. Notably, the analysis, including FMI and VAT mass, was limited to a smaller subsample who had undergone DXA measurement. There is a risk that those who did not undergo DXA might have been significantly different than those in the sub-sample, resulting in selection bias. Furthermore, despite underweight being one of the important risk factors of pre-frailty and frailty, we had to exclude underweight individuals from our analyses due to low numbers, thus, being unable to conclude anything regarding this group.

We used education as the objective measure of social position. Although education is a widely recognized indicator, we acknowledge that social position encompasses various factors beyond education, such as income, occupation, wealth and residential area, which were not addressed in our study. Nevertheless, education plays a pivotal role in shaping an individual’s opportunities for improved health and well-being [58, 59]. Our categorisation of education into two groups: ≤ 10 years, which included primary/partly secondary education and > 10 years, which included a combination of upper secondary and tertiary levels, provided us with a simplified framework for comparing individuals in lower social position with those in relatively higher social position based on their educational attainment. However, this might have obscured the variability within the > 10 years of education group.

Growing evidence highlights the importance of complementing objective measures of socioeconomic position with subjective assessments, emphasizing a close connection between subjective perceptions and health outcomes [60–62]. So, with the acknowledgement that education might be confounded by age and not entirely capture one’s perception of social position and its influence on health-related choices and decisions [60, 63], we used subjective perception of social position as well. The measure that we have utilized in the present study captures an individual’s own assessment of their occupation’s social position, rather than how society perceives it [62]. However, owing to low representation, individuals who reported their social position as low were not included in our analysis. As a result, we could only compare the effect of high versus medium subjective social position on the association between adiposity and pre-frailty. It is possible that the association between adiposity and pre-frailty could have differed significantly for those perceiving their social position as low, and our inability to assess this limits our conclusion and its generalisability to populations with different distributions of social position. Furthermore, we also acknowledge the potential for selection bias in our study resulting from the overall lower participation rate of individuals with low social position in the Tromsø7 study [64].

A major limitation of the present study is its cross-sectional design. Thus, we could not assess how specific social strata may have influenced the risk of developing pre-frailty in individuals with varying levels of adiposity over the course of their lives. Although we focused primarily on pre-frailty as the main outcome, it would have been valuable to explore both pre-frailty and frailty as outcomes. This would have allowed us to compare whether social variables impact the association between adiposity measures and different stages of physical frailty similarly or distinctly. Unfortunately, this was not possible owing to the low prevalence of frailty in our study sample. Nevertheless, an understanding of the factors linked with pre-frailty is equally, if not more, important, because it presents a timely opportunity to prevent or delay the onset of frailty and promote healthy ageing. Our assessment of frailty status was based on Fried et al.’s frailty phenotype definition [1]. While the performance-based frailty indicators (grip strength and walking speed) utilised in our study have been objectively measured in line with standardised protocols, the self-reported indicators (exhaustion, low physical activity, and unintentional weight loss) are prone to information bias. According to the Fried et. al’s definition, individuals with one or two frailty indicators are categorised as pre-frail. However, it is worth noting that most of the pre-frail population in our study exhibited a frailty score of 1, and among them, low physical activity was the most common indicator (50%), followed by unintentional weight loss (22%). There is a probability that pre-frail individuals with only low physical activity as a frailty indicator in our study might have been sedentary but otherwise healthy individuals. Furthermore, we were unable to estimate the magnitude of unintentional weight loss, which could have led to the overestimation of pre-frail individuals with this indicator. Additionally, although we adjusted for several confounding factors available in our study, the potential for residual confounding from unobserved variables remains. These limitations should be accounted for while interpreting the findings from this study.

We consistently observed an increased risk of pre-frailty among women and men with excess adiposity, with only slight variation by difference in social position. At normal BMI levels, we observed higher pre-frailty risk among individuals with lower social position compared with those with higher social position. This underscores the importance of preventing excess adiposity to prevent frailty and promote healthy ageing among everyone while recognizing the distinct vulnerabilities of individuals with low social position. Furthermore, comprehensive longitudinal studies considering the effect of life course socioeconomic position on the association between obesity and pre-frailty, as well as frailty, are needed to validate and expand on these findings.

### Electronic supplementary material

Below is the link to the electronic supplementary material.

Supplementary Material 1

## Data Availability

The data that support the findings of this study are available from the Tromsø Study, but restrictions apply to the availability of these data, which were used under license for the current study, and so are not publicly available. The data can be made available from the Tromsø Study upon application to the Tromsø Study Data and Publication Committee. The legal restriction on data availability has been set by the Tromsø Study Data and Publication Committee to control data sharing, including the publication of data sets with the potential of reverse identification of de-identified sensitive participant information. The links to the main questionnaires used in different surveys of the Tromsø Study can be found on the Tromsø Study’s website (https://uit.no/research/tromsostudy). A detailed information on the variables collected can be found in Helsedata (https://helsedata.no/en/variables/?datakilde=K_TR&page=search).

## Abbreviations

BMI: Body mass index
CI: Confidence interval
DXA: Dual-energy x-ray absorptiometry
FMI: Fat mass index
OR: Odds ratio
RD: Risk difference
RR: Risk ratio
SD: Standard deviation
SPPB: Short physical performance battery
VAT: Visceral adipose tissue
WC: Waist circumference
