# Supplementary material for: Does the association between adiposity measures and pre-frailty among older adults vary by social position? Findings from the Tromsø study 2015/2016
Source: BMC Public Health. 2024 May 31;24:1457. doi: 10.1186/s12889-024-18939-3 (PMC11140933; doi:10.1186/s12889-024-18939-3)
Supplement: Supplementary file 1 — Supplementary Material 1 [file 12889_2024_18939_MOESM1_ESM.docx]

Eligible participants who meet the age criteria (≥65 years):

**5,874**

Excluded: **21**

(missing information on all five frailty indicators in Tromsø7)

Attended Tromsø7:

**21,083**

Not available for analysis: **14**

Excluded: **15,195**

(<65 years)

Eligible study sample:

**5,853**

Excluded: **54**

(missing information on BMI or WC in Tromsø7)

Eligible study sample:

**5,799**

Excluded: **55**

(frail participants)

Eligible study sample:

**5,744**

Excluded: **5**

(missing information on both social variables of interest: education and subjective social status)

Eligible study sample:

**5,739**

Individuals with information on DXA-derived adiposity measures: **2,191**

Supplementary Figure 1 Flowchart displaying participants’ inclusion and exclusion.

**Supplementary Table 1 Comparison between Fried et al.’s criteria for frailty and modified frailty indicators used in the present study**

| **Frailty** | **Fried et al.** | **Tromsø7** |
| --- | --- | --- |
| **Exhaustion** | Questions from the Center for Epidemiologic Studies Depression Scale:  (a) I felt that everything I did was an effort  (b) I could not get going  How often in the last week did you feel this day?  0 = Rarely or none of the time (<1 day)  1 = Some or a little of the time (1–2 days)  2 = A moderate amount of time (3–4 days)  3 = Most of the time  **Exhausted: ‘A moderate amount of time (3–4 days)’ or ‘Most of the time’** | Hopkins Symptom Checklist (HSCL-10):  During the last week, have you experienced that everything is a struggle?  1 = No complaint  2 = Little complaint  3 = Pretty much  4 = Very much  **Exhausted: Category 3 and 4 ‘Pretty much’ or ‘Very much’** |
| **Physical activity** | Minnesota Leisure Time Activity Questionnaire asking about walking, chores (moderately strenuous), mowing the lawn, raking, gardening, hiking, jogging, biking, exercise, cycling, dancing, aerobics, bowling, golf, singles, tennis, racquetball, calisthenics, swimming  The kcal/week expended was calculated using a standardized algorithm. Lowest 20% were identified, resulting in following cut-off for frailty:  Men: <383 kcal of physical activity/week  Women: <270 kcal of physical activity/week | Describe your exercise and physical exertion in leisure time over the last year (Saltin–Grimby scale):  1 = Reading, watching TV/screen or other sedentary activity  2 = Walking, cycling, or other forms of exercise at least 4 hours a week  3 = Participation in recreational sports, heavy gardening, snow shovelling, etc. at least 4 hours a week  4 = Participation in hard training or sports competitions, regularly several times a week  **Low physical activity level: Category 1 ‘Reading, watching TV/screen or other sedentary activity’** |
| **Weight loss** | In the last year, have you lost more than 10 pounds (4.5 kg) unintentionally (not due to dieting or exercise)?  **Frail: ‘Yes’** | Have you involuntarily lost weight during the last 6 months? (Malnutrition Universal Screening Tool)  0 = No  1 = Yes  **Lost weight: ‘Yes’** |
| **Grip strength** | Measured by Jamar dynamometer (kg)  Maximal strength in dominant hand (3 trials)  Stratified by sex and BMI quartiles. Lowest 20% were identified, resulting in the following cut-off for frailty:  **Men Cut-off for grip strength (kg) criterion for frailty**  BMI <24 ≤29 kg  BMI 24.1–26 ≤30 kg  BMI 26.1–28 ≤30 kg  BMI >28 ≤32 kg  **Women**  BMI <23 ≤17 kg  BMI 23.1–26 ≤7.3 kg  BMI 26.1–29 ≤18 kg  BMI >29 ≤21 kg | Measured by Jamar dynamometer (kg); strongest measurement from three trials in each hand  Stratified by sex and BMI quartiles as per Fried’s definition:  **Men Cut-off for grip strength (kg) criterion for frailty**  BMI <24 ≤29 kg  BMI 24.1–26 ≤30 kg  BMI 26.1–28 ≤30 kg  BMI >28 ≤32 kg  **Women**  BMI <23 ≤17 kg  BMI 23.1–26 ≤17.3 kg  BMI 26.1–29 ≤18 kg  BMI >29 ≤21 kg |
| **Walking speed** | Time to walk (seconds) 15 feet at usual pace stratified by sex and height (sex-specific cut-off at medium height): Lowest 20% were identified, resulting in the following cut-off for frailty:  **Men Cut-off for walking speed criterion for frailty**  Height ≤173 cm ≥7 s  Height >173 cm ≥6 s  **Women**  Height <159 cm ≥7 s  Height >159 cm ≥6 s | SPPB: Short Physical Performance Battery – walking test  Fastest of two times (seconds) to walk 4 m stratified by sex and height according to Fried's sex-specific cut-off. Converted to feet from metres.  **Men Cut-off for walking speed criterion for frailty**  Height ≤173 cm ≥7 s  Height >173 cm ≥6 s  **Women**  Height <159 cm ≥7 s  Height >159 cm ≥6 s |
| **Frailty status** | Frailty score:  0 = robust  1–2 = pre-frail  ≥3 = frail | Frailty score:  0 = robust  1–2 = pre-frail |

|  |  |  | **Supplementary Table 2 Participants' characteristics by pre-frailty scores** | | | | | | | | | | | | | | | | | | | |
| --- | --- | --- | --- | --- | --- | --- | --- | --- | --- | --- | --- | --- | --- | --- | --- | --- | --- | --- | --- | --- | --- | --- |
|  | | | |  | **Total (n=5739)** | | | | |  | **Women (n=2945)** | | | | |  | **Men (n=2794)** | | | | | |
|  | | | | **Frailty status** | | | |  |  | **Frailty status** | | | |  |  | **Frailty status** | | | |  |  | |
|  | | | | **Robust** | | **Pre-frail (score 1)** | **Pre-frail**  **(score 2)** | | **p-value** | **Robust** | | **Pre-frail**  **(score 1)** | **Pre-frail**  **(score 2)** | | **p-value** | **Robust** | | **Pre-frail**  **(score 1)** | **Pre-frail**  **(score 2)** | | | **p-value** |
|  | | | | **% (n)** | | **% (n)** | **% (n)** | |  | **% (n)** | | **% (n)** | **% (n)** | |  | **% (n)** | | **% (n)** | **% (n)** | | |  |
|  | | | | **72.9 (4182)** | | **23.1 (1328)** | **4.0 (229)** | |  | **71.3 (2099)** | | **23.8 (702)** | **4.9 (144)** | |  | **74.5 (2083)** | | **22.4 (626)** | **3.1 (85)** | | |  |
| **Age, mean (SD)** | | | | 71.6 (5.6) | | 73.5 (6.6) | 74.6 (6.2) | | 0.000^a^ | 71.5 (5.6) | | 73.5 (6.6) | 74.5 (6.2) | | 0.000^a^ | 71.6 (5.5) | | 72.7 (6.0) | 74.7 (6.4) | | | 0.015^a^ |
| **Smoking status** | | | |  | |  |  | |  |  | |  |  | |  |  | |  |  | | |  |
| Current smokers | | | | 9.3 (382) | | 14.7 (193) | 14.7 (33) | |  | 10.4 (214) | | 13.8 (95) | 14.8 (21) | |  | 8.2 (168) | | 15.8 (98) | 14.5 (12) | | |  |
| Former smokers | | | | 52.6 (2170) | | 52.4 (687) | 49.3 (111) | | 0.000 | 46.9 (969) | | 47.1 (325) | 47.9 (68) | | 0.05 | 58.3 (1201) | | 58.3 (362) | 51.8 (43) | | | 0.000 |
| Never | | | | 38.1 (1573) | | 32.9 (431) | 36.0 (81) | |  | 42.7 (883) | | 39.1 (270) | 37.3 (53) | |  | 33.5 (690) | | 25.9 (161) | 33.7 (28) | | |  |
| **Alcohol** | | | |  | |  |  | |  |  | |  |  | |  |  | |  |  | | |  |
| Frequent drinkers | | | | 31.0 (1281) | | 23.5 (308) | 17.2 (39) | |  | 26.7 (552) | | 19.1 (132) | 14.8 (21) | |  | 35.3 (729) | | 28.3 (176) | 21.2 (18) | | |  |
| Infrequent drinkers | | | | 57.9 (2394) | | 60.8 (798) | 58.6 (133) | | 0.000 | 59.5 (1229) | | 58.5 (404) | 52.1 (74) | | 0.000 | 56.3 (1165) | | 63.5 (394) | 69.4 (59) | | | 0.002 |
| Never/ Abstaining | | | | 11.1 (459) | | 15.7 (206) | 24.2 (55) | |  | 13.8 (286) | | 22.4 (155) | 33.1 (47) | |  | 8.4 (173) | | 8.2 (51) | 9.4 (8) | | |  |
| **Married/Cohabiting** | | | |  | |  |  | |  |  | |  |  | |  |  | |  |  | | |  |
| Married/ Cohabiting | | | | 69.6 (2911) | | 63.5 (843) | 57.2 (131) | | 0.000 | 58.7 (1232) | | 51.8 (364) | 51.4 (74) | | 0.003 | 80.6 (1679) | | 76.5 (479) | 67.1 (57) | | | 0.002 |
| Living alone | | | | 30.4 (1271) | | 36.5 (485) | 42.8 (98) | |  | 41.3 (867) | | 48.2 (338) | 48.6 (70) | |  | 19.4 (404) | | 23.5 (147) | 32.9 (28) | | |  |
| **Self-perceived health** | | | |  | |  |  | |  |  | |  |  | |  |  | |  |  | | |  |
| Good | | | | 69.0 (2844) | | 46.8 (613) | 25.7 (58) | | 0.000 | 68.2 (1404) | | 45.6 (314) | 21.1 (30) | | 0.000 | 69.8 (1440) | | 48.2 (299) | 33.3 (28) | | | 0.000 |
| Poor | | | | 31.0 (1278) | | 53.2 (697) | 74.3 (168) | |  | 31.8 (654) | | 54.4 (375) | 78.9 (112) | |  | 30.2 (624) | | 51.8 (322) | 66.7 (56) | | |  |
| **Comorbidity** | | | |  | |  |  | |  |  | |  |  | |  |  | |  |  | | |  |
| No comorbidity | | | | 61.5 (2562) | | 48.1 (634) | 42.1 (96) | | 0.000 | 61.1 (1275) | | 47.6 (331) | 40.6 (58) | | 0.000 | 61.9 (1287) | | 48.6 (303) | 44.7 (38) | | | 0.000 |
| Comorbidity | | | | 38.5 (1602) | | 51.9 (685) | 57.9 (132) | |  | 38.9 (811) | | 52.4 (365) | 59.4 (85) | |  | 38.1 (791) | | 51.4 (320) | 55.3 (47) | | |  |
| **Education** | | | |  | |  |  | |  |  | |  |  | |  |  | |  |  | | |  |
| >10 years of education | | | | 61.4 (2547) | | 51.3 (675) | 45.8 (105) | | 0.000 | 54.8 (1142) | | 42.5 (295) | 36.1 (52) | | 0.000 | 68.0 (1405) | | 61.2 (380) | 62.3 (53) | | | 0.005 |
| ≤10 years of education | | | | 38.6 (1603) | | 48.7 (641) | 54.2 (124) | |  | 45.2 (942) | | 57.5 (400) | 63.9 (92) | |  | 32.0 (661) | | 38.8 (241) | 37.7 (32) | | |  |
| **Subjective social status** | | | |  | |  |  | |  |  | |  |  | |  |  | |  |  | | |  |
| High | | | | 41.8 (1675) | | 37.5 (458) | 34.1 (73) | |  | 33.4 (666) | | 31.0 (196) | 27.9 (36) | |  | 50.2 (1009) | | 44.6 (262) | 43.5 (37) | | |  |
| Medium | | | | 53.2 (2128) | | 56.2 (685) | 63.1 (135) | | 0.002 | 59.7 (1188) | | 61.3 (388) | 69.8 (90) | | 0.074 | 46.7 (940) | | 50.6 (297) | 52.9 (45) | | | 0.061 |
| Low | | | | 5.0 (199) | | 6.3 (77) | 2.8 (6) | |  | 6.9 (137) | | 7.7 (49) | 2.3 (3) | |  | 3.1 (62) | | 4.8 (28) | 3.5 (3) | | |  |
| **Adiposity measures** | | | |  | |  |  | |  |  | |  |  | |  |  | |  |  | | |  |
| **BMI, kg/m^2^, mean(SD)** | | | | *27.1 (3.9)* | | *28.2 (5.0)* | *28.3 (6.0)* | | 0.000^a^ | *26.8 (4.4)* | | *28.1 (5.5)* | *28.7 (6.5)* | | 0.000^a^ | *27.3 (3.5)* | | *28.3 (4.5)* | *27.7 (5.1)* | | | 0.000^a^ |
| Underweight | | | | 0.5 (22) | | 1.1 (15) | 2.2 (15) | |  | 1.0 (20) | | 2.0 (14) | 2.1 (3) | |  | 0.1 (2) | | 0.2 (1) | 2.4 (2) | | |  |
| Normal | | | | 30.7 (1284) | | 23.8 (316) | 26.6 (61) | | 0.000 | 35.4 (743) | | 25.1 (176) | 25.7 (37) | | 0.000 | 26.0 (541) | | 22.4 (140) | 28.2 (24) | | | 0.000 |
| Overweight | | | | 48.3 (2020) | | 42.3 (562) | 35.8 (82) | |  | 43.0 (902) | | 39.6 (278) | 33.3 (48) | |  | 53.7 (1118) | | 45.4 (284) | 40.0 (34) | | |  |
| Obesity | | | | 20.5 (856) | | 32.8 (435) | 35.4 (81) | |  | 20.6 (434) | | 33.3 (234) | 38.9 (56) | |  | 20.2 (422) | | 32.1 (201) | 29.4 (25) | | |  |
| **WC, mean (SD)** | | | | *96.0 (11.8)* | | *99.3 (13.6)* | *99.5 (15.0)* | | 0.000^a^ | *91.7 (11.7)* | | *95.3 (13.7)* | *97.0 (15.1)* | | 0.000^a^ | *100.4 (10.1)* | | *103.9(12.0)* | *103.7(13.4)* | | |  |
| Normal | | | | 23.0 (963) | | 16.5 (219) | 18.8 (43) | |  | 17.9 (376) | | 12.8 (90) | 13.9 (20) | |  | 28.2 (587) | | 20.6 (129) | 27.1 (23) | | |  |
| Moderately high | | | | 27.9 (1166) | | 22.2 (295) | 16.2 (37) | | 0.000 | 23.5 (493) | | 18.4 (129) | 13.9 (20) | | 0.000 | 32.3 (673) | | 26.5 (166) | 20.0 (17) | | | 0.000 |
| High | | | | 49.1 (2053) | | 61.3 (814) | 65.0 (149) | |  | 58.6 (1230) | | 68.8 (483) | 72.2 (104) | |  | 39.5 (823) | | 52.9 (331) | 52.9 (45) | | |  |
| **n= 2193** | | | |  | |  |  | |  | **n= 1282** | |  |  | |  | **n= 911** | |  |  | | |  |
| **FMI, kg/m^2^, mean (SD)** | | | | *9.2 (3.2)* | | *10.2 (3.9)* | *11.1 (4.8)* | | 0.000^a^ | *10.4 (3.2)* | | *11.6 (3.9)* | *12.6 (4.8)* | | 0.000^a^ | *7.6 (2.5)* | | *8.2 (2.8)* | *8.3 (3.5)* | | | 0.002^a^ |
| **FMI tertiles** | | | |  | |  |  | |  |  | |  |  | |  |  | |  |  | | |  |
| Low (T1) | | | |  | |  |  | |  | 36.2 (342) | | 27.1 (75) | 18.6 (11) | |  | 35.7 (243) | | 25.5 (51) | 33.3 (10) | | |  |
| Medium (T2) | | | |  | |  |  | |  | 35.3 (334) | | 28.9 (80) | 22.0 (13) | | 0.000 | 32.3 (220) | | 38.0 (76) | 26.7 (8) | | | 0.089 |
| High (T3) | | | |  | |  |  | |  | 28.5 (270) | | 44.0 (122) | 59.3 (35) | |  | 32.0 (218) | | 36.5 (73) | 40.0 (12) | | |  |
| **VAT, g, mean (SD)** | | | | *1277 (782)* | | *1460 (906)* | *1499 (956)* | | 0.000^a^ | *983 (594)* | | *1154 (711)* | *1292 (823)* | | 0.000^a^ | *1685 (827)* | | *1885 (976)* | *1906 (1079)* | | | 0.002^a^ |
| **VAT tertiles** | | | |  |  |  |  | |  |  |  |  |  | |  |  |  |  |  | | |  |
| Low (T1) | | | |  | |  |  | |  | 35.4 (335) | | 28.8 (80) | 22.0 (13) | |  | 35.3 (241) | | 26.5 (53) | 36.7 (11) | | |  |
| Medium (T2) | | | |  | |  |  | |  | 34.4 (325) | | 30.6 (85) | 30.5 (18) | | 0.002 | 33.8 (231) | | 34.5 (69) | 13.3 (4) | | | 0.011 |
| High (T3) | | | |  | |  |  | |  | 30.2 (286) | | 40.6 (113) | 47.5 (28) | |  | 30.9 (211) | | 39.0 (78) | 50.0 (15) | | |  |
| Values are mean values (standard deviations) or percentages (numbers).  P-value: Chi^2^ test for categorical variables; ^a^ P-value: One way anova for continuous variables. BMI, body mass index; FMI, fat mass index; SD, standard deviation; VAT, visceral adipose tissue; WC, Waist circumference. T1: first tertile; T2: second tertile; T3: third tertile. BMI categories WC categories Underweight: <18.5 kg/m^2^  Normal: men ≤94cm; women ≤80cm Normal: 18.5–24.9 kg/m^2^ Moderately high: men 95–102cm; women 81–88cm  Overweight: 25.0–29.9 kg/m^2^ High: men >102cm; women> 88cm  Obesity: ≥30 kg/m^2^  FMI categories VAT categories Low (T1): women <9.07 kg/m^2^; men <6.55 kg/ m^2^  Low (T1): women <694g; men <1286g Medium (T2): women 9.07–11.99 kg/m^2^; men 6.55–8.82 kg/m^2^  Medium (T2): women 694–1214g; men 1286–2045g High (T3): women >11.99 kg/m^2^ ; men >8.82 kg/m^2^  High (T3): women >1214g; men >2045g | | | | | | | | | | | | | | | | | | | | | | |

| **Supplementary Table 3 Participant’s adiposity measures by measures of social status** | | | | | | | | |
| --- | --- | --- | --- | --- | --- | --- | --- | --- |
|  | **Education** | | | | **Subjective social status** | | | |
|  | **Women (*n*= 2,923)** | | **Men (*n*= 2,772)** | | **Women (*n*= 2,564)** | | **Men (*n*= 2,590)** | |
|  | **>10 years** | **≤10 years** | **> 10 years** | **≤10 years** | **High** | **Medium** | **High** | **Medium** |
|  | **% (*n*)** | **% (*n*)** | **% (*n*)** | **% (n)** | **% (*n*)** | **% (*n*)** | **% (*n*)** | **% (*n*)** |
|  | **50.9 (1,489)** | **49.1 (1,434)** | **66.3 (1,838)** | **33.7 (934)** | **35.0 (898)** | **65.0 (1,666)** | **50.5 (1,308)** | **49.5 (1,282)** |
| **BMI** |  |  |  |  |  |  |  |  |
| Normal | 37.5 (552) | 28.2 (399) | 26.5 (487) | 22.8 (213) | 37.6 (333) | 32.2 (531) | 25.5 (333) | 25.5 (326) |
| Overweight | 40.8 (600) | 43.7 (619) | 51.7 (948) | 51.1 (476) | 38.8 (343) | 43.6 (719) | 51.5 (672) | 51.5 (660) |
| Obesity | 21.7 (206) | 28.1 (397) | 21.8 (400) | 26.1 (243) | 23.6 (209) | 24.2 (398) | 23.0 (299) | 23.0 (295) |
| *P* value | <0.001 |  | 0.02 |  | 0.02 |  | 0.99 |  |
|  |  |  |  |  |  |  |  |  |
| **WC** |  |  |  |  |  |  |  |  |
| Normal | 20.5 (305) | 12.4 (178) | 27.1 (499) | 25.2 (235) | 20.4 (183) | 15.4 (257) | 26.7 (349) | 26.3 (337) |
| Moderately high | 22.6 (336) | 21.0 (301) | 31.1 (571) | 29.7 (277) | 21.2 (190) | 22.9 (381) | 31.3 (409) | 29.6 (379) |
| High | 56.9 (613) | 66.6 (955) | 41.8 (768) | 45.2 (422) | 58.4 (525) | 61.7 (1028) | 42.0 (385) | 44.1 (566) |
| *P* value | <0.001 |  | 0.22 |  | 0.01 |  | 0.52 |  |
|  | ***n*= 1,279** | | ***n*= 907** | | ***n*= 1,137** | | ***n*= 850** | |
| **FMI tertiles** |  |  |  |  |  |  |  |  |
| Low (T1) | 40.1 (265) | 26.3 (162) | 33.8 (200) | 32.5 (102) | 39.7 (161) | 31.1 (227) | 34.1 (141) | 33.5 (146) |
| Medium (T2) | 32.1 (212) | 34.5 (213) | 35.2 (208) | 30.2 (95) | 29.3 (119) | 35.3 (258) | 30.7 (127) | 34.9 (152) |
| High (T3) | 27.8 (184) | 39.2 (242) | 31.0 (183) | 37.3 (117) | 31.0 (126) | 33.6 (246) | 35.2 (146) | 31.6 (138) |
| *P* value | <0.001 |  | 0.13 |  | 0.011 |  | 0.371 |  |
|  |  |  |  |  |  |  |  |  |
| **VAT mass tertiles** |  |  |  |  |  |  |  |  |
| Low (T1) | 41.5 (275) | 24.6 (152) | 33.6 (199) | 33.3 (105) | 37.9 (154) | 31.6 (231) | 32.1 (133) | 35.0 (153) |
| Medium (T2) | 31.0 (205) | 36.1 (223) | 32.8 (194) | 33.6 (106) | 29.8 (121) | 34.7 (254) | 33.7 (140) | 32.7 (143) |
| High (T3) | 27.5 (182) | 39.2 (242) | 33.6 (199) | 33.1 (104) | 32.3 (131) | 33.7 (247) | 34.2 (142) | 32.3 (141) |
| *P* value | <0.001 |  | 0.96 |  | 0.07 |  | 0.65 |  |
| Values are percentages (numbers).  *P* value: χ^2^ test for categorical variables.  BMI, body mass index; FMI, fat mass index; SD, standard deviation; VAT, visceral adipose tissue; WC, waist circumference.  T1: first tertile; T2: second tertile; T3: third tertile.  **BMI categories WC categories**  Underweight: <18.5 kg/m^2^  Normal: women ≤80 cm; men ≤94 cm  Normal: 18.5–24.9 kg/m^2^ Moderately high: women 81–88 cm; men 95–102 cm  Overweight: 25.0–29.9 kg/m^2^ High: women> 88cm; men >102 cm  Obesity: ≥30 kg/m^2^  **FMI categories VAT categories**  Low (T1): women <9.1 kg/m^2^; men <6.5 kg/ m^2^ Low (T1): women <694 g; men <1,286 g  Medium (T2): women 9.1–12.0 kg/m^2^; men 6.5–8.8 kg/m^2^  Medium (T2): women 694–1,214 g; men 1,286–2,045 g  High (T3): women >12.0 kg/m^2^; men >8.8 kg/m^2^  High (T3): women >1,214g; men >2,045g | | | | | | | | |
|  | | | | | | | | |

**Supplementary Table 4 Association between adiposity measures and pre-frailty by education**

|  | **Women (*n*= 2,886)** | | **Men (*n*= 2,767)** | |
| --- | --- | --- | --- | --- |
|  | **RR (95% CI)** | **RD (95% CI)** | **RR (95% CI)** | **RD (95% CI)** |
| **BMI and education** |  |  |  |  |
| Normal BMI & >10 years of education | Ref | Ref | Ref | Ref |
| Normal BMI & ≤10 years of education | **1.49 (1.17–1.90)** | **0.08 (0.03–0.14)** | 1.12 (0.86–1.47) | 0.03 (−0.04 to 0.09) |
| Overweight & >10 years of education | **1.28 (1.01–1.62)** | 0.03 (−0.01 to 0.07) | 1.02 (0.82–1.26) | 0.02 (−0.02 to 0.06) |
| Overweight & ≤10 years of education | **1.36 (1.08–1.72)** | **0.06 (0.01–0.11)** | 1.06 (0.84–1.34) | 0.02 (−0.03 to 0.07) |
| Obesity & >10 years of education | **1.92 (1.51–2.44)** | **0.15 (0.10–0.21)** | **1.56 (1.25–1.95)** | **0.11 (0.06–0.17)** |
| Obesity & ≤10 years of education | **1.91 (1.53–2.41)** | **0.19 (0.13–0.24)** | **1.56 (1.21–1.98)** | **0.13 (0.06–0.20)** |
|  | **Women (*n*= 2,923)** | | **Men (*n*= 2,772)** | |
| **WC and Education** |  |  |  |  |
| Normal WC & >10 years of education | Ref | Ref | Ref | Ref |
| Normal WC & ≤10 years of education | 1.25 (0.91–1.71) | 0.04 (−0.03 to 0.11) | 0.98 (0.73–1.32) | −0.01 (−0.07 to 0.05) |
| Moderate WC & >10 years of education | 0.90 (0.64–1.24) | −0.02 (−0.06 to 0.03) | 0.96 (0.75–1.23) | −0.01 (−0.05 to 0.03) |
| Moderate WC & <10 years of education | 1.26 (0.93–1.69) | 0.06 (−0.01 to 0.12) | 1.12 (0.86–1.46) | 0.02 (−0.04–0.08) |
| High WC & >10 years of education | **1.29 (1.00–1.67)** | **0.05 (0.01–0.10)** | **1.37 (1.11–1.69)** | **0.07 (0.03–0.12)** |
| High WC & <10 years of education | **1.38 (1.06–1.79)** | **0.09 (0.04–0.14)** | **1.42 (1.13–1.79)** | **0.10 (0.04–0.15)** |
|  | **Women (*n*= 1,278)** | | **Men (*n*= 905)** | |
| **FMI and education** |  |  |  |  |
| Low FMI & >10 years of education | Ref | Ref | Ref | Ref |
| Low FMI & ≤10 years of education | 1.45 (0.98–2.13) | 0.06 (−0.02 to 0.14) | 1.02 (0.65–1.61) | −0.03 (−0.11 to 0.05) |
| Medium FMI & >10 years of education | 1.37 (0.94–1.99) | 0.04 (−0.03 to 0.11) | 1.32 (0.91–1.91) | 0.05 (−0.03 to 0.13) |
| Medium FMI & ≤10 years of education | 1.13 (0.77–1.68) | 0.01 (−0.06 to 0.08) | 1.31 (0.86–1.99) | 0.04 (−0.06 to 0.14) |
| High FMI & >10 years of education | **1.82 (1.27–2.63)** | **0.12 (0.04–0.20)** | 1.16 (0.78–1.72) | 0.02 (−0.06 to 0.10) |
| High FMI & ≤10 years of education | **2.01 (1.43–2.82)** | **0.18 (0.10–0.26)** | **1.57 (1.07–2.30)** | **0.11 (0.004–0.21)** |
|  | **Women (*n*= 1,279)** | | **Men (*n*= 907)** | |
| **VAT mass and education** |  |  |  |  |
| Low VAT mass & >10 years of education | Ref | Ref | Ref | Ref |
| Low VAT mass & ≤10 years of education | 1.28 (0.89–1.83) | 0.04 (−0.04 to 0.12) | 1.02 (0.66–1.58) | −0.03 (−0.11 to 0.06) |
| Medium VAT mass & >10 years of education | 1.07 (0.74–1.54) | −0.01 (−0.07 to 0.05) | 1.08 (0.73–1.59) | 0.00 (−0.07 to 0.08) |
| Medium VAT mass & ≤10 years of education | 1.18 (0.85–1.65) | 0.02 (−0.05 to 0.09) | 1.24 (0.81–1.88) | 0.04 (−0.07 to 0.14) |
| High VAT mass & >10 years of education | **1.45 (1.02–2.04)** | **0.08 (0.00–0.16)** | 1.27 (0.88–1.83) | 0.04 (−0.05 to 0.12) |
| High VAT mass & ≤10 years of education | **1.47 (1.08–2.02)** | **0.10 (0.02–0.18)** | **1.51 (1.04–2.20)** | 0.09 (−0.01 to 0.19) |
| CI, confidence interval; RD: risk difference; RR: risk ratio. | | | | |
| Adjusted for age, smoking status, alcohol intake status, comorbidity, marital/cohabitation status and self-perceived health.  BMI, body mass index; FMI, fat mass index; SD, standard deviation; VAT, visceral adipose tissue; WC, waist circumference.  T1: first tertile; T2: second tertile; T3: third tertile.  **BMI categories WC categories**  Underweight: <18.5 kg/m^2^  Normal: women ≤80 cm; men ≤94 cm  Normal: 18.5–24.9 kg/m^2^ Moderately high: women 81–88 cm; men 95–102 cm  Overweight: 25.0–29.9 kg/m^2^ High: women> 88 cm; men >102 cm  Obesity: ≥30 kg/m^2^  **FMI categories VAT categories**  Low (T1): women <9.1 kg/m^2^; men <6.5 kg/ m^2^  Low (T1): women <694 g; men <1,286 g  Medium (T2): women 9.1–12.0 kg/m^2^; men 6.5–8.8 kg/m^2^  Medium (T2): women 694–1,214 g; men 1,286–2,045 g  High (T3): women >12.0 kg/m^2^; men >8.8 kg/m^2^  High (T3): women >1,214 g; men >2045 g | | | | |

**Supplementary Table 5 Association between adiposity measures and pre-frailty by subjective social status**

|  | **Women (*n*=2533)** | | **Men (*n*=2585)** | |
| --- | --- | --- | --- | --- |
|  | **RR (95% CI)** | **RD (95% CI)** | **RR (95% CI)** | **RD (95% CI)** |
| **BMI and subjective social status** |  |  |  |  |
| Normal BMI & high social status | Ref | Ref | Ref | Ref |
| Normal BMI & medium social status | 1.01 (0.78–1.31) | −0.01 (−0.05 to 0.04) | 1.30 (0.98–1.73) | **0.06 (0.01–0.12)** |
| Overweight & high social status | 1.08 (0.82–1.43) | 0.00 (−0.05 to 0.05) | 1.11 (0.85–1.45) | 0.03 (−0.01 to 0.08) |
| Overweight & medium social status | 1.14 (0.90–1.46) | 0.03 (−0.02 to 0.08) | 1.18 (0.90–1.54) | 0.05 (0.00–0.09) |
| Obesity & high social status | **1.70 (1.29–2.22)** | **0.15 (0.07–0.23)** | **1.96 (1.49–2.58)** | **0.18 (0.12–0.25)** |
| Obesity & medium social status | **1.60 (1.25–2.05)** | **0.14 (0.08–0.20)** | **1.62 (1.22–2.14)** | **0.12 (0.06–0.18)** |
|  | **Women (*n*= 2,564)** | | **Men (*n*= 2,590)** | |
| **WC and subjective social status** |  |  |  |  |
| Normal WC & high social status | Ref | Ref | Ref | Ref |
| Normal WC & medium social status | 0.92 (0.65–1.31) | −0.02 (−0.08 to 0.05) | 1.14 (0.84–1.54) | 0.02 (−0.03 to 0.08) |
| Moderate WC & high social status | 0.96 (0.66–1.41) | 0.01 (−0.07 to 0.08) | 1.05 (0.78–1.42) | 0.00 (−0.04 to 0.05) |
| Moderate WC & medium social status | 0.89 (0.64–1.23) | −0.02 (−0.09 to 0.04) | 1.16 (0.86–1.55) | 0.03 (−0.03 to 0.08) |
| High WC & high social status | 1.18 (0.87–1.59) | 0.04 (−0.02 to 0.10) | **1.57 (1.21–2.04)** | **0.10 (0.05–0.15)** |
| High WC & medium social status | 1.20 (0.90–1.60) | 0.06 (0.00–0.12) | **1.46 (1.13–1.90)** | **0.09 (0.03–0.14)** |
|  | **Women (*n*= 1,137)** | | **Men (*n*= 850)** | |
| **FMI and subjective social status** |  |  |  |  |
| Low FMI & high social status | Ref | Ref | Ref | Ref |
| Low FMI & medium social status | 0.78 (0.51–1.18) | −0.04 (−0.11 to 0.04) | 1.24 (0.76–2.01) | 0.02 (−0.07 to 0.09) |
| Medium FMI & high social status | 0.98 (0.63–1.52) | −0.04 (−0.12 to 0.04) | 1.53 (0.96–2.46) | 0.06 (−0.03 to 0.15) |
| Medium FMI & medium social status | 1.07 (0.74–1.55) | 0.02 (−0.06 to 0.10) | 1.43 (0.88–2.32) | 0.06 (−0.03 to 0.16) |
| High FMI & high social status | 1.38 (0.93–2.04) | 0.09 (−0.02 to 0.19) | 1.42 (0.88–2.29) | 0.04 (−0.05 to 0.12) |
| High FMI & medium social status | **1.50 (1.06–2.13)** | **0.12 (0.03–0.21)** | 1.54 (0.96–2.47) | 0.08 (−0.02 to 0.17) |
|  | **Women (*n*= 1,138)** | | **Men (*n*= 852)** | |
| **VAT mass and subjective social status** |  |  |  |  |
| Low VAT mass & high social status | Ref | Ref | Ref | Ref |
| Low VAT mass & medium social status | 0.94 (0.64–1.40) | −0.01 −0.09 to 0.07) | 1.35 (0.83–2.20) | 0.05 (−0.04 to 0.14) |
| Medium VAT mass & high social status | 1.08 (0.70–1.66) | −0.01 (−0.10 to 0.08) | 1.49 (0.91–2.42) | 0.06 (−0.03 to 0.15) |
| Medium VAT mass & medium social status | 1.05 (0.73–1.51) | −0.002 (−0.08 to 0.07) | 1.12 (0.67–1.88) | 0.01 (−0.08 to 0.10) |
| High VAT mass & high social status | 1.26 (0.85–1.87) | 0.05 (−0.05 to 0.15) | 1.46 (0.90–2.36) | 0.04 (−0.05 to 0.13) |
| High VAT mass & medium social status | 1.39 (0.97–1.98) | **0.09 (0.01–0.18)** | **1.75 (1.09–2.80)** | **0.12 (0.01–0.22)** |
| CI, confidence interval; RD: risk difference; RR: risk ratio. | | | | |
| Adjusted for age, smoking status, alcohol intake status, comorbidity, marital/cohabitation status and self-perceived health.  BMI, body mass index; FMI, fat mass index; SD, standard deviation; VAT, visceral adipose tissue; WC, waist circumference.  T1: first tertile; T2: second tertile; T3: third tertile.  **BMI categories WC categories**  Underweight: <18.5 kg/m^2^  Normal: women ≤80 cm; men ≤94 cm  Normal: 18.5–24.9 kg/m^2^ Moderately high: women 81–88 cm; men 95–102 cm  Overweight: 25.0–29.9 kg/m^2^ High: women> 88 cm; men >102 cm  Obesity: ≥30 kg/m^2^  **FMI categories VAT categories**  Low (T1): women <9.1 kg/m^2^; men <6.5 kg/ m^2^  Low (T1): women <694 g; men <1,286 g  Medium (T2): women 9.1–12.0 kg/m^2^; men 6.5–8.8 kg/m^2^ Medium (T2): women 6 94–1,214 g; men 1,286–2,045 g  High (T3): women >12.0 kg/m^2^; men >8.8 kg/m^2^  High (T3): women >1,214 g; men >2,045 g | | | | |

**Supplementary Table 6 Frequency of individual frailty indicators at Tromsø7 (n = 5739) ^a^**

|  | **Pre-frailty**  **(score 1 or 2)**  **(*n* = 1557) ^b^** | **Pre-frailty**  **(score 1)**  **(*n* = 1328) ^c^** |
| --- | --- | --- |
| Frailty indicators  Exhaustion, *n* = 5467  Slow walking speed, *n* = 3778  Low grip strength, *n* = 3782  Weight loss, *n* = 5540  Low physical activity, *n* = 5302 | n (%)  151 (2.7%)  188 (4.9%)  242 (6.4%)  390 (7.0%)  815 (15.4%) | n (%)  94 (1.7%)  111 (2.9%)  164 (4.3%)  297 (5.4%)  662 (12.5%) |

^a^Primary analytic sample. ^b^Total pre-frail participants . ^c^ Pre-frail participants with frailty score 1.

n (%) Percentage prevalence calculated among participants with valid data on the specific frailty components
